# Factors Influencing Engagement in a Digital Substance Use Prevention Program: Qualitative Study Using the Capability, Opportunity, Motivation Model and Theoretical Domains Framework

**DOI:** 10.2196/64861

**Published:** 2025-08-14

**Authors:** Nikolai Kiselev, Rebecca Schaffner, Zsofia Csepregi, Andreas Wenger, Raquel Paz Castro, Severin Haug

**Affiliations:** 1 Swiss Research Institute for Public Health and Addiction (ISGF), University of Zurich, Konradstrasse 32, Zurich, 8005, Switzerland, 41 444481174

**Keywords:** SmartCoach, program engagement, digital intervention, substance use prevention, COM-B model, theoretical domains framework, engagement optimization, adolescent, teenager, substance use, drug use, engagement, qualitative study, training program, prevention, mobile health, mHealth, text, cigarette, cannabis, social support, social influences

## Abstract

**Background:**

Digital interventions are promising for the prevention of substance use in young people. However, engagement with these interventions is often insufficient, and their full potential remains unrealized. Given the established link between engagement in digital interventions and their effectiveness, understanding user factors that influence involvement in eHealth and mobile health (mHealth) interventions is essential.

**Objective:**

This study aimed to identify factors influencing user engagement in a mobile phone–based life skills training program for substance use prevention among adolescents and to collect suggestions for program optimization.

**Methods:**

A qualitative study was conducted with 171 participants of the mHealth prevention SmartCoach (Pathmate Technologies) program. The program provided individualized text messages to foster life skills for 4 months and proved to be effective in preventing the onset of cigarette and cannabis use. Semistructured phone interviews were conducted with program participants to explore factors associated with program engagement and to gather suggestions for program optimization. Interviews were recorded, transcribed, and analyzed using thematic analysis with both inductive and deductive coding. The capability, opportunity, and motivation model of behavior change (COM-B) model and Theoretical Domains Framework (TDF) were used to assess behavioral influences.

**Results:**

Key factors positively influencing program engagement included the timing of text messages, social influences and support, engaging and helpful content, and rewards (points and prizes). Conversely, barriers to engagement were identified as forgetfulness, short response time limits, limited time resources, lack of interest, concerns related to personal disclosure, and difficulty identifying with the challenge task type (posting). Suggestions for optimization included implementing reminders, providing better guidance for using tips, allowing personalization of message timing and content, extending time limits for tasks, and reducing the concerns related to personal disclosure.

**Conclusions:**

The study confirms the critical role of timing, content relevance, and social support in enhancing engagement with digital interventions. Specific recommendations for optimizing the SmartCoach program were derived, highlighting the importance of reminders, personalization, and addressing concerns related to personal disclosure.

## Introduction

### Background

The consumption of tobacco, alcohol, and cannabis poses serious health risks and can lead to addiction. According to the World Health Organization (WHO), millions of individuals die each year from the consequences of consuming these substances [1]. Despite the known risks, usage rates remain high. Across Europe, there are significant variations in consumption, with almost 10% of the population consuming alcohol daily, over a quarter being smokers, and around 1.3% using cannabis [2-5].

The prevalence of substance use increases sharply between the ages of 11 and 15 years [6]. In Switzerland, the lifetime prevalence of alcohol use has been documented to increase from 22% among 11-year-old boys to 70% among 15-year-old boys and from 11% in 11-year-old girls to 69% in 15-year-old girls [7]. The proportion of students who reported having smoked cigarettes at least once in their life similarly increased from 6% to 35% in 11-year-old boys versus 15-year-old boys and from 2% to 30% in 11-year-old girls versus 15-year-old girls, respectively. On average, in the Canton of Zurich, alcohol consumption begins at around 15 years of age, tobacco at 14.6 years, and cannabis at 15.6 years [8]. Therefore, early and midadolescence appear to be a critical period for introducing substance use prevention interventions. School settings are particularly suitable, as they facilitate delivery and access, especially within compulsory education systems.

Life skills–based intervention programs to prevent substance use delivered in the school have been effective at preventing the onset of specific substances [9-11] and at decreasing problematic substance use [12]. However, their implementation and dissemination in schools present serious challenges [13], as teachers and other professionals need the time, motivation, knowledge, and skills to deliver the program [14].

Digital interventions have great potential to overcome these obstacles. Excluding development costs, mobile health (mHealth) interventions have an extensive reach at low cost and offer the ability to deliver uniquely personalized content automatically, which can be accessed at any time and anywhere [15].

However, a major challenge in delivering digital preventive measures is the lack of control over adolescents’ engagement with the intervention. Several reviews on digital interventions to promote mental health [16,17] or to prevent substance use [15] in young people point to relatively low levels of user engagement. Regular use of mHealth programs is often the exception rather than the rule, which is subsumed under the term “engagement crisis” [18].

There is a wide body of evidence showing that higher engagement (ie, a higher number of user interactions within a program) in a prevention program leads to better outcomes for participants [19-22]. The results of the mobile phone–based SmartCoach (Pathmate Technologies) program to prevent substance use in adolescents also revealed this relationship. For instance, participants with a higher engagement rate in the intervention showed a lower likelihood of tobacco consumption in the follow-up survey. Furthermore, participants who actively participated in almost all program components reported the highest increase in well-being [23]. In addition to user engagement during the program, several studies emphasize the positive effects of active youth involvement in the development of prevention messages and program content [24,25]. These findings support the idea that engagement should not only be seen as a behavioral outcome but also as a process of cocreation, which can contribute to stronger identification with the program and its goals.

The challenge to increase participant engagement in digital preventive intervention programs is increasingly discussed from both scientific and practical perspectives; however, there is a lack of generalizable approaches that show how engagement in existing programs can be systematically improved [18].

Promising starting points for the identification of factors related to program engagement and behavior change are provided by the capability, opportunity, and motivation model of behavior change (COM-B) model [23,26] and the Theoretical Domains Framework (TDF) [27].

According to the COM-B model, behavior (eg, program engagement) arises from the interaction between the individual’s psychological and physical capacity to engage in the activity (eg, literacy skills), the physical and social opportunity (eg, peer support), and automatic and reflective motivation (eg, rewards). The model offers a high-level yet structured perspective to identify which components may need to be addressed to achieve behavior change. It is particularly suitable for designing and analyzing health interventions, as it helps to distinguish whether a lack of engagement, for instance, stems from skill deficits (capability), contextual barriers (opportunity), or limited intention or drive (motivation).

The TDF is a synthesis of 33 theories and 128 psychological constructs [27]. It includes 14 domains (eg, knowledge and environmental resources) that can be mapped under the 3 components of the COM-B model [27,28]. The framework was developed to support the systematic identification of behavioral influences and to guide the development of theory-informed interventions. In qualitative research, the TDF is frequently used to structure deductive coding, as it allows researchers to break down complex behavioral phenomena into well-defined, theory-based domains.

The combination of these 2 models has been widely applied in research. For example, Szinay et al [29] used the COM-B and TDF models to investigate experiences with and reasons for using a broad range of health and well-being apps through interviews. Rosário et al [30] used the COM-B and TDF framework to identify factors influencing the implementation of screening and brief interventions for alcohol consumption in primary care.

### Research Aim

This study aimed to identify factors derived from the COM-B and TDF framework that might increase engagement in the SmartCoach program, an mHealth intervention to prevent substance use by fostering life skills in adolescents. Specifically, this qualitative study aimed to address the following research questions: (1) What factors positively or negatively influence engagement with the SmartCoach program? and (2) What suggestions do participants offer to optimize the program?

## Methods

### Overview

A qualitative study was conducted based on free-listing interviews. In the course of the first research question, an exploratory and explanatory approach was pursued, following the framework proposed by Guest et al [31]. The second research question was exploratory. Thematic analysis was used to analyze the interviews, using NVivo 14 software (Lumivero). To assess the generalizability of the results, a nonresponder analysis was also conducted. This paper was prepared following the Journal Article Reporting Standards for Qualitative Research (JARS), as proposed by the American Psychological Association [32], to ensure transparent and rigorous reporting.

### Intervention Program and Setting

The fully automated, internet- and SMS text messaging–based intervention program SmartCoach is based on social cognitive theory and addresses self-management skills, social skills, and substance use resistance skills [33,34]. Participants receive 1-4 SMS text messages per week during the 4-month intervention period. Active program engagement was encouraged through interactive features such as quiz questions, messages, and picture contests, and the integration of a friendly competition with prizes, where users collect credits with each interaction. The efficacy of the program was tested in a cluster randomized controlled trial with 1759 students from 89 Swiss secondary and upper secondary school classes [34,35]. Of these, 1473 (83.7%) students participated in both the program and the respective study; their mean age was 15.4 years. On average, program participants responded to about half of the prompted activities, with adolescents who did not drink in a problematic manner and those with higher educational levels showing the highest usage rates [23].

Longer-term results at the 18-month follow-up showed a reduction in smoking prevalence in the intervention group compared to controls. However, no significant effect was observed for at-risk drinking. No significant moderators of the primary outcomes were observed; that is, the program’s effectiveness was independent of age, sex, migration background, and school level.

While participant access and engagement in the previous efficacy study [34,35] may have been influenced by the assessment-only control condition, this study was conducted under more naturalistic conditions, without a control group, and focused on identifying factors associated with program uptake and engagement [36].

### Sample

For the abovementioned study focusing on program uptake and engagement, we recruited a total of 476 students from 28 secondary and upper secondary school classes in the Swiss cantons of Zurich and Aargau between September and December 2022. Recruitment was conducted within a school workshop about the topic of stress, with the option for further participation in the SmartCoach program offered at the end of the workshop. Of these, 315 (66.2%) students were willing to participate in the SmartCoach program and received weekly text messages for 4 months [36]. This subsample constitutes the population for the results presented in this qualitative study.

### Interviewers

All interviews were conducted by 2 female psychology master’s students from the University of Zurich (in their final year). The interviewers had been involved in interview preparation since the recruitment phase for the intervention. Besides previous interviewing experience in other research projects, they were additionally trained in practicing free-listing interviews with this study’s target population by the project’s senior researcher. In preparation for the data collection, 2 half-day workshops were held with the interviewers, during which the methodological approach and procedures were discussed in detail. In addition, the interviewers conducted practice interviews both within the research team and with 3 adolescents, each of whom was not part of the SmartCoach program, to refine their interviewing skills and ensure clarity and appropriateness of the questions.

### Interview Guide

To collect the data, an interview guide was developed in collaboration with researchers from the SmartCoach project. The questions were free-listing, allowing respondents to provide open-ended answers without being constrained by the question [37]. The interview guide comprised 22 questions, mostly about general program participation, including reasons for high and low involvement (first part) and optimization suggestions based on experiential feedback (second part). The last part consisted of a think-aloud task, during which personal stressors and possible coping strategies were queried (for possible future development of the program). This part of the interview guide is neither included in the present work nor published elsewhere. It is important to note that participants (50:50) were asked questions alternating between the first and second or the first and last parts of the interview guide.

A total of 5 pilot interviews were conducted to validate the interview guide. Based on these, the interview guide was discussed, and several questions were revised. During the pilot interviews, it was noticed that responses, particularly in the first part, might be influenced by social desirability. In response, a section explaining the interview’s goals was added to the introduction, emphasizing that the aim was not to evaluate SmartCoach positively but to answer the questions as honestly as possible.

### Recruitment

All interviews were conducted between January 2023 and May 2023 by phone in German, encompassing both regional dialects and standard German. The prevalent call timeframe was on weekdays, typically from approximately 4 to 7 PM, chosen to optimize accessibility for students during those hours.

### Procedure

After the interventional part of the SmartCoach program was finished, participants received an SMS text message reminding them about the postprogram interview in the subsequent days. In addition, this message informed the students that they would receive CHF 10 (approximately US $10) after completing the interview. The participants’ list was split randomly between the interviewers. After reaching out, the participants were informed about the interview aims. Inclusion criteria included willingness to participate in the interview and consent to the audio recording. Subsequently, the interviews started.

Participants who could not be reached during the first call attempt were called for 2 more times on other days. If these attempts were unsuccessful, a text message was sent requesting appointment suggestions for the interview. Further contact attempts were discontinued if there was no response to this message either. In total, a maximum of 4 contact attempts were made: 3 calls and 1 text message. All audio recordings of the interviews were subsequently transcribed.

### Analysis

The interviews were analyzed using the thematic analysis method [38]. NVivo 14 software was used for data analysis. Following the approach outlined by Gale et al [39] and Braun et al [40], the subsequent steps detail the process undertaken during the application of thematic analysis. Subsequently, codes were created using both an inductive and deductive approach. Inductive codes emerged directly from the data, while deductive codes followed the structure of the COM-B and TDF, as also presented in Table 1 in the “Results” section (Braun et al [40] and Castleberry and Nolen [41]). The research team approved the coding framework after several rounds of iterative adaptation. After establishing the final coding framework, the interrater reliability was calculated based on 5 transcripts (k=0.88 indicating excellent agreement). Thereafter, transcripts were split between 2 coders. Finally, 2 senior researchers additionally cross-checked the final coding results. Furthermore, it is important to mention that it was possible to allocate a statement to different codes simultaneously.

**Table 1.** Number of sources citing promoting and inhibiting factors.

COM-B^a^ model components and TDF^b^ domains	Source		
	Promoting factor, n	Inhibiting factor, n	Sum, n
Capability			
Physical			
Skills	2	0	2
Psychological			
Skills	9	6	15
Knowledge	3	9	12
Memory, attention, and decision process	7	44	51
Behavior regulation	1	21	22
Opportunity			
Physical			
Environmental context and resources	44	74	118
Social			
Social influences	38	3	41
Motivation			
Reflective			
Social identity	3	0	3
Beliefs about capabilities	3	1	4
Beliefs about consequences	44	2	46
Optimism	17	7	24
Goals	9	2	11
Intentions	7	13	20
Automatic			
Emotions	60	32	92
Optimism	0	2	2
Social identity	0	20	20
Reinforcement	20	3	23

a COM-B: capability, opportunity, and motivation model of behavior change.

b TDF: Theoretical Domains Framework.

Conducting a nonresponder analysis was essential to assess the generalizability of the results. This analysis examined the differences between the students who participated in the interview and those who did not. The following variables were of interest: age, gender, number of interactions in the SmartCoach program, type of school (secondary vs upper secondary school), and migration background (defined as the student themselves, their father, or their mother being from a non-German-speaking country vs none). For the analysis of the variables age and the number of interactions in the SmartCoach program, independent sample *t* tests were conducted for each. For the analysis of the variables age, school type (secondary vs upper secondary school), and migration background, the Pearson chi-square test for associations was applied. The analyses were conducted in IBM SPSS Statistics version 29.

### Ethical Considerations

Ethical approval for this study was granted on April 17, 2022, by the Ethics Committee of the Faculty of Philosophy at the University of Zurich (22.2.15). In Switzerland, parental consent is not required for participation in prevention programs such as SmartCoach for adolescents aged 14 years or older. All participants provided active digital informed consent by registering for the program. Study participants completed the screening procedure and baseline assessment anonymously. No names were collected; instead, participants chose nicknames. Password protection and Secure Sockets Layer encoding were used to ensure the privacy and safety of data transfer. Study participants received an incentive of CHF 10 (approximately US $12) for taking part in the follow-up survey.

## Results

### Participant Characteristics

Out of the 315 students (who participated in SmartCoach) contacted, only 184 students were reached, and interviews could be conducted with 171 (54.3%) students. Another 13 (4.1%) students were reached but did not consent to the interview, all citing a lack of interest. A majority of the interviewed students were female (107/171, 62.6%). There were no students who identified themselves aside from traditional binary genders. Their age ranged from 13 to 19 years, with an average age of 15.4 (SD 1.1) years. In total, 99 (57.9%) students attended secondary school, while 72 (42.1%) students were in upper secondary school. For 84 (49.1%) students, at least 1 person in the household was from a non-German-speaking country. At the same time, the other half of the participants (85/171) were considered native-born since no person in the household was from a non–German-speaking country. Two students did not provide information about their potential migration background. The shortest interview lasted 5:46 minutes, and the longest lasted 17:44 minutes. The average interview duration was 11:33 minutes.

### Nonresponder Analysis

Participants did not differ from nonresponders regarding age (*t*_312_=0.281; *P*=.78), school type (*χ*^2^_1_=.206; *P*=.65; n=315) and migration background (*χ*^2^_1_=2.17; *P*=.14; n=315). However, significant differences regarding the number of interactions with the program (*t*_312_=−10.63; *P*<.001) and gender (*χ*^2^_1_=5.6; *P*=.02; n=315) were found.

### Factors Influencing Engagement

Every element of the TDF and COM-B model was identified during the interviews, but the frequencies varied strongly among the factors, as presented in Table 1. A total of 7 factors were mentioned fewer than 18 times by each interviewee. They are briefly summarized in the last subchapter, whereas other factors are discussed in more detail below, depending on their role and possible positive, negative, or rather ambivalent (not mostly positive or negative) influencing factors. 

### Factors Influencing Engagement Positively

Based on the participants’ answers, 3 notably mentioned factors (“beliefs about consequences,” “social influences,” and “reinforcement”) were identified as positively influencing engagement in the SmartCoach program. The TDF domain “beliefs about consequences” (within the reflective part of motivation in the COM-B model) was mentioned 44 times, making it the second most frequently mentioned positively influencing factor. In most cases, participants reported intrinsic expectations such as:

*I think that can help me.*

[ID 197]

*[...] that you can also learn from it.*

[ID 137]

*[...] I thought if I participate, it might help me, and that’s why I just tried it.*

[ID 32]

This was often related to the topic of stress, particularly in the school context, as the following quote shows:

*For example, in times of stress, I participated in everything […] and SmartCoach helps me a lot. *

[ID 117]

The TDF domain “social influences” (as part of the social subcategory within the COM-B model) was mentioned almost as frequently as “beliefs about consequences,” with 38 sources. However, only 2 sources of social influence were discussed. On the one hand, students stated that their peers motivated them a lot, and they participated because others were also involved, as the following 2 quotes show:

*Because of my colleague, we were always together outside, and when she participated, I thought, I'll join in too.*

[ID 315]

*[…] my colleagues participated, and if my colleagues participate, then I participate too.*

[ID 122]

On the other hand, the information and recruiting session in the classes was mentioned as an influencing factor that positively impacted engagement. Students found it informative and motivating:

*So, this woman came to our school and explained everything, and from that moment, it really motivated me to participate, and that’s why I answered all the questions.*

[ID 213]

*Yes [the reason was] the person who came into the classroom, the energy from that person.*

[ID 281]

Another factor strengthening participation (in the SmartCoach program) was incentives (students had the opportunity to receive CHF 10 (approximately US $12) after participating in the program and to win prizes through a periodic lottery among program participants). The 20 statements regarding them, such as “simply because of the prize*”* [ID 205] or because “money motivates” [ID 66], were allocated to the TDF domain “reinforcement” within the automatic subcategory of motivation in the COM-B model. However, the reinforcement was not limited to material incentives, as the following 2 quotes show:

*I liked the credit system quite a bit, that drove me, I really wanted to reach the 100 credits.*

[ID 195]

*And, on the other hand, it was an incentive that you got credits through participation.*

[ID 62]

In contrast, these 3 domains were mentioned only a couple of times as inhibiting factors (a maximum of 3 times).

### Factors Influencing Engagement Negatively

Next to the strengthening factors, 3 notable factors (“memory, attention, and decision process,” “behavioral regulation,” and “social identity”) were identified as negatively influencing engagement in the SmartCoach program. The TDF domain “memory, attention, and decision processes” within the psychological component of the capability of the COM-B model was the second most frequently (n=44) cited domain that may have had inhibiting effects on engagement. Statements such as “it slipped my mind” [ID 142] and “Sometimes I forgot to respond*”* [ID 296] were predominant. Most students acknowledged receiving a text message and, in some cases, even read it. However, they often forgot to participate afterward, as expressed by ID 122:

*I saw it, and then I had to leave immediately and didn’t see it again, and then it got lost or forgotten.*

[ID 122]

Overall, the key issue here was that students frequently forgot to respond.

Another TDF domain—“behavioral regulation”—was mentioned frequently (n=21) as an inhibitor and was allocated to the same psychological component of the capability of the COM-B model. Two main themes were prominent in this domain. First, some students mentioned that they did not even look at text messages because they do not use SMS text messaging or have turned off notifications, so the message was not perceived at all:

*[...] because I have turned off the notification for text messages on my phone, so I don’t receive notifications for text messages.*

[ID 131]

Second, there was often a discussion about the time limit imposed on some interactive content by SmartCoach (the opportunity to respond was limited to 2 hours in some cases (eg, the picture contests). Many were too late to respond:

*[...] except for the pictures and videos. Because I always saw them too late. Because in the evening, I don’t always check my phone. It was until eight o’clock, and then I didn’t check it anymore. [...] I just always saw the contests too late. *

[ID 108]

A further group of statements (n=20) allocated to the TDF domain “social identity” within the automatic category of motivation of the COM-B model was strongly related to the issue of posting within the contests. Many interviewees could not identify themselves with uploading personal-related pictures or statements, as was asked in the program contests, as shown in the following quotes:

*Posting is not really my thing*.

[ID 129]

*I'm not the type to post*.

[ID 161]

*Because I don't post much on social media myself, I don't have any social media except WhatsApp, and that’s generally not my area, so I didn't really participate.*

[ID 49]

In contrast, these 3 domains were mentioned very rarely as strengthening factors (a maximum of up to 7 times).

### Factors Influencing Engagement in an Ambivalent Way

In addition to TDF domains that were reported as positive or negative influencing factors, 2 domains were reported as contradictory. On the one hand, 44 statements allocated to the TDF domain “environmental context and resources” within the physical subcategory of the COM-B model’s opportunity might be interpreted as facilitators for program participation. On the other hand, according to 74 sources, this domain was reported to have the strongest inhibiting influence on engagement among all interviews. In this context, the majority of statements addressed time constraints, which were observed to be the most controversial issues, as presented in Textbox 1.

Textbox 1. **Controversial quotes related to time issues were stated by interviewees.**

**Time as a strengthening factor:**

- “So the messages always came at five o’clock on Tuesday or so. We were always on our way home from school and always had time to respond.” [ID 108]
- “Most of the time it was easy because the message just came, and I didn’t have anything else to do.” [ID 225]

**Time as an inhibiting factor:**

- “It was just always at an inconvenient time.” [ID 142]
- “Because the text messages always came at a certain time when you might not have needed it.” [ID 44]

Other statements closely related to the time matter were the issue of overload with other duties, as the following quote shows:

*I have enough stress because of football and school, and when I'm tired in the evening from school or because I have stress with the coach, then I consider the text message rather unimportant and annoying. I think, 'Oh, not again the text message,' and then I swipe it away. Not meant to be rude, but in that moment, I just needed time for myself.*

[ID 263]

Furthermore, the statements indicated that for many, the lack of internet was also a reason for their lower participation. Some needed the internet to respond to the text message for free, and others could not open the links to interactive parts of SmartCoach without the internet:

*I received the text message, and I wasn’t at home, then I couldn’t open the link because I didn’t have Internet. Then I came home and forgot about it, and then it was too late, which I found unfortunate.*

[ID 291]

Another group of statements that received ambivalent feedback was related to the TDF domain “emotions” from the automatic subcategory within the component “motivation” of the COM-B model. In contrast to the previous domain, 60 people believed it was a factor positively impacting program engagement. Fun, interest in the program, and perception of the content as exciting were particularly prominent. Many mentioned that it was “interesting to see what comes as a response” [ID 53] and found the program “really good” [ID 250]. A respondent said:

*Honestly, I found it still exciting, answering the questions was fun, thinking about what you are interested in, and reading the tips.*

[ID 165]

And another respondent expressed:

*Because I found it super cool, I always looked forward to it; it was also my favorite task in the whole study. I thought it was cool to show what helps me and to see what others have said, what target groups were also participating in the study.*

[ID 223]

However, for 32 interviewees, this domain inhibited engagement. Furthermore, concerning the frequency of mentioning, this domain was named the second most frequent inhibiting factor. In the absolute majority of answers, the reasons were lack of interest and lack of desire to participate, as the following 2 statements present:

*So if the topics didn't interest me 100%, then I skipped them.*

[ID 152]

*I just received the messages and didn't look at them closely anymore. Actually, there wasn't a real reason; I had seen it over time and didn't feel like participating anymore.*

[ID 93]

### Factors Influencing Engagement Mentioned Rarely

From the rest of the domains that were rarely reported (under 18 times), 2 domains were named in sum (promoting + inhibiting influence) by over 10% of the sources: the TDF domains “Optimism” (n=24) and “Intentions” (n=20)—both within the automatic part of the component “Motivation” in the COM-B model. However, the statements for both were somewhat ambiguous. They might not be allocated firmly to promoting or inhibiting factors, making it impossible to consider them a silent influencing factor for a specific minority.

### Suggestions Regarding Program Adjustments

The responses regarding possible adjustment of the program (these questions formed the second part of the interview and were asked only to half of the respondents [Methods section]) were categorized into 2 notable subcodes with the themes of “Text Messages” (n=43) and “Contests” (n=29). Most of the suggestions for optimizing text messages were related to reminder messages (n=19) and the timing of the messages (n=30). However, a closer analysis of the suggestions associated with the time of the messages showed that the opinion regarding better messaging time split almost equally among 3 proposals: SMS text message on the weekend (n=15), in the late evening (n=14), or the morning (n=10). Besides a couple of statements about the number of contests (more [n=5] vs less [n=2]), participants who talked about the issue of contests recommended increasing the time limit to react to the contest (n=19).

The same picture showed the replies to the question about the communication medium. One-half of the participants rated SMS text messaging positively (n=73), calling SMS text messaging “*uncomplicated*” [ID 195], *“great”* [ID 19], *“clear”* [ID 197], and *“practical”* [ID 225]. However, another half was convinced that better communication methods, instead of SMS text messaging, should be used (n=69). Among them, WhatsApp (Meta) was mentioned most frequently (n=20). In addition, it should be noted that almost half of this subgroup added that even though better communication methods exist, SMS text messaging still seemed acceptable (n=36).

Overall, participants evaluated the length of the program as well as the number and frequency of the messages mostly positively (n=60 and n=72), with a considerable number of individuals wishing for a shorter program (n=29), compared with a few people wanting a more extended program (n=11). Only a few individuals wished for more (n=14) or fewer (n=6) SMS text messages during the program.

In addition to the two main subcodes presented above (“Text Messages” and “Contests”), two topics within the sum of over 30 residual statements regarding program adjustment (n=32) were mentioned more than twice and therefore warrant further discussion. Within this residual subcategory, personalization (n=5) was most related to the issue that the messages seemed to be too “robotic,” as the following example shows:

*The response sometimes seemed almost programmed, with the 'Too bad' and all, sometimes you almost felt like you were talking to a robot, […] maybe making it more personal when there is capacity for it.*

[ID 223]

Another topic mentioned 5 times (n=5) within the residual category was related to the tips or suggestions that sounded simple but were challenging to implement. A few participants wished for more detailed guidance on how to implement the tips. The remaining suggestions regarding program adjustments were strongly heterogeneous and sometimes opposed to one another.

Furthermore, it needs to be noted that a significant part of the participants reported positive statements (n=57), such as *“*very exciting*”* [ID 165], *“*very helpful*”* [ID 107], and *“*understandable” [ID 285] regarding the program, and at the same time, also addressed the issue of potential adjustment. The following statement summarizes the positive answers as follows:

*I found it good, you filled out at the beginning which topics are important to you, and there were always questions/tips in that area, so I found it good.*

[ID 255]

### Feedback on the Program’s Content

Since the program consisted of 3 major blocks (self-management skills, social skills, and substance use resistance skills), interviewees were asked about feedback on these 3 superior topics. Regarding self-management skills, most responses were positively allocated to the subcodes “helpful” (n=29) and “interesting” (n=32). At the same time, 20 individuals considered the topic less relevant to them. Many expressed that they were already familiar with the tips and strategies or did not personally experience a personal necessity, making them less relevant, as the following quote shows:

*So, I didn't really need the tips for school stress; I'm doing well in school. They were good, but I didn't need them.*

[ID 193]

Only 5 people stated that the tips related to social skills were not helpful. The majority considered the tips concerning social skills as “interesting” (n=34) or helpful (n=17). At the same time, 26 interviewees believed that the statements were not relevant to them, as the following quotation highlights:

*[…] I don't really have problems dealing with others, so I couldn't really use them […].*

[ID 171]

Answers related to substance use resistance skills mirrored the same pattern as the previous 2 content areas: 37 persons considered the topic “interesting,” and another 23 described it as “important to discuss.” In contrast, 23 interviewees thought the topic was irrelevant to them.

## Discussion

### Principal Findings

This study aimed to identify factors derived from the COM-B model and the TDF influencing engagement in an mHealth program for adolescent substance use prevention. The findings provide valuable insights into promoting and inhibiting factors affecting engagement, accessed by means of structured qualitative interviews, offering practical implications for enhancing the effectiveness of digital interventions. In the following section, the results are first discussed individually in relation to their specific outcomes and then synthesized to highlight overarching patterns, including contradictory findings and their implications for future implementation.

### Promoting Factors for Engagement

Qualitative analyses revealed that the following 5 TDF domains most commonly positively influenced program participation: “environmental context and resources,” “social influences,” “emotions,” “reinforcement,” and “beliefs about consequences.” Specifically, the following factors were perceived as promoting engagement: the right timing of text messages, the involvement of other classmates, collaborative content processing, and the informational session during recruitment. In addition, engaging intervention content, rewards, and incentives in the form of points and prizes, and the inherent curiosity of participants about the program content further enhanced engagement.

These findings align with previous research emphasizing the importance of timely and relevant content in digital interventions [42,43]. The role of social support and interactive content in promoting engagement is well-documented [43-45]. The effectiveness of rewards and incentives in motivating participation has also been supported by studies on digital health interventions [44].

### Inhibiting Factors Regarding Engagement

In terms of inhibiting factors regarding engagement, the following 5 TDF domains were identified as particularly relevant: memory, attention, decision process, behavior regulation, environmental context and resources, emotion, and social identity. Specifically, the following contents were found to inhibit engagement: forgetting to participate, inactivity on the communication channel and the time limit, lack of time resources and internet access, concerns related to personal disclosure, lack of interest and desire, and a lack of identification with a topic.

These barriers are consistent with challenges noted in previous studies. Forgetfulness and timing issues have been highlighted as common obstacles in digital interventions [43,44]. The need for personalization and flexibility in intervention delivery is crucial to address diverse user needs and preferences [42,46-48]. Concerns related to personal disclosure, particularly regarding not wishing to share personal or even any information, remain a significant barrier to digital health engagement [42,43].

### Domains That Can Have Both Inhibiting and Facilitating Influences

Interestingly, some factors, such as the “Environmental context and resources” and “Emotion” domains, had both promoting and inhibiting influences. Positive emotions, such as finding the content fun and interesting, facilitated engagement, while negative emotions, such as a lack of interest or feeling overwhelmed, inhibited participation. Similarly, the timing and context of text messages could either support or hinder engagement depending on individual circumstances.

### Implications for the Further Development of the SmartCoach Program

To maintain the successful elements of the SmartCoach program, it is essential to preserve the passionate and engaging introduction sessions conducted in schools, as these have proven to significantly boost initial interest and participation. In addition, the use of SMS text messages or comparable messaging systems as a communication medium should be retained, given its widespread acceptance and effectiveness in reaching adolescents. These components have demonstrated their value in fostering engagement and should remain integral parts of the program.

At the same time, based on the findings of the study, several optimization suggestions concerning the SmartCoach intervention program were derived. First, to increase engagement regarding the usage of program content (reading the messages, answering them, watching additional topic-related materials [eg, videos]), the introduction of a text message reminder for participation is suggested. This can help reduce the likelihood of forgetting to participate, even if the text message is received at an inconvenient time. This solution would address the “memory, attention, and decision process” aspect frequently mentioned in the context of inhibited acting. The use of reminders to enhance participation is also found in the literature [46,48]. However, it is important to ensure that sending reminders via text message does not exceed the tolerated number of messages. Intrusive notifications can trigger negative emotions or reactance, which, in turn, can harm engagement [44].

Closely connected to this aspect is also the reported fact related to the “Environmental context and resources” that participation was often forgotten because the text message arrived at an inconvenient time for many participants. In such cases, responding was postponed and then forgotten. Therefore, changing the text message’s timing may be meaningful in reaching the users when they are receptive and available to engage with the intervention [49]. A significant number of respondents expressed a preference for the weekend as a better time. However, opinions vary regarding the time of day. In contrast, the timing of the text message was also found to be among the facilitating factors, indicating that many participants were satisfied with the timing. Here, it was a clear aspect that might be both inhibiting and promoting activity depending on the individual situation. A solution could be stronger personalization: allowing participants to choose or define an individual time slot for receiving text messages during the initial survey. Personalization as a strategy to increase engagement is well-represented in the literature [42,46-48].

An issue that also frequently led to low participation was the time limit for completing picture or message contests. Participants’ suggestions in this regard were to increase the time limit, with the majority desiring an extension from the original 2 hours to several days. It is important to note that while it may be sensible to increase the time limit to this extent, there is also a risk that this could lead to an increased likelihood of forgetting to participate. Therefore, in the context of extending the time limit, the consideration of reminders should also be taken into account. In combination with a later reminder, an extension for a few hours might also be considered, giving participants a longer time slot, such as an entire evening, and allowing them to react later in case of other activities during the evening. This optimization suggestion is specific to SmartCoach and has not been confirmed by the existing literature.

In addition to the options for addressing the inhibiting factors discussed above, 3 further domains—“behavior regulation,” “social identity,” and “emotions”—were frequently mentioned as barriers but appear to be more challenging to address directly through program design alone. These domains are closely tied to individual preferences, values, and psychological traits, making one-size-fits-all solutions less feasible.

For example, behavior regulation, such as turning off all notifications, is typically a matter of personal autonomy. A possible response could be to inform participants at the start of the program about the importance of notifications and recommend enabling them during participation. Still, this measure may have a limited effect, as SMS text message notifications cannot easily be customized for individual programs such as SmartCoach, and participants might deliberately choose to restrict digital interruptions as part of their general phone usage behavior.

Similarly, social identity-related barriers, such as discomfort with posting or participating in peer group challenges, often reflect deeper self-concepts or social positioning. Removing these program components entirely could have unintended negative consequences for other users by weakening positively perceived elements such as “beliefs about consequences,” which emerged as a key engagement facilitator in this study. As a possible optimization, offering participants the choice to engage in such content at the beginning of the program—similar to the possibility of choosing the preferred time for receiving messages as described above—could help accommodate individual preferences. In addition, providing alternative task types for those who are reluctant to take part in group interactions or offering clearer information about who can see uploaded content may further reduce discomfort and increase perceived safety. Maintaining a flexible structure that allows optional participation in such tasks may help to protect both individual comfort and the overall program effect.

In addition, the domain of emotions—although mentioned in both promoting and inhibiting ways—was described more frequently as a positive contributor to engagement. Therefore, attempting to influence this factor through design changes should be cautiously approached. Interventions that try to evoke specific emotions or suppress negative ones risk being perceived as manipulative and could reduce user trust or trigger reactance [50,51]. Thus, maintaining an emotionally balanced, user-sensitive communication tone may be the most appropriate strategy. The domains “reinforcement,” “beliefs about consequences,” and “social influences” from the TDF were identified in this study as positively associated with participant engagement. From this perspective, it is essential to maintain and further strengthen program elements that foster these perceptions. For instance, financial incentives such as the small monetary reward at the end of the program served as a reinforcement mechanism; statements such as “*everyone participated, so I did too*” reflected the impact of peer dynamics; and beliefs that the program would be useful or bring something new were key motivators for engagement.

However, an overly strong emphasis on these aspects might have unintended consequences. For example, excessive reliance on social conformity (“everyone else is doing it”) could undermine intrinsic motivation and lead to disengagement among those who value autonomy. This may also relate to the previously discussed aspect of social identity, particularly in cases where individuals prefer not to share personal content or engage in group-based activities. Similarly, if reinforcement mechanisms such as financial incentives are overused, participants may perceive the program as externally controlling rather than personally meaningful. A careful balance is therefore needed to preserve the motivational power of these domains without inducing dependency, loss of authenticity, or reactance.

Since, as already mentioned, not all topics addressed during the SmartCoach program were relevant to everyone, there were suggestions for additional topics that participants might find more interesting. One possible solution might be applying the topics’ selection for the individual challenges to the regular messages or adapting the messages more closely to the preselected topics for the individual challenges. However, one must consider whether participants might misappropriate the possibility of strong personalization of topics; ultimately, SmartCoach remains a program for addiction prevention and should not serve as entertainment.

Finally, a few participants also wished for a more detailed explanation of how to implement the tips and strategies. Providing a clear user guide for using an intervention is also discussed in previous literature as a facilitating factor for engagement [44]. However, the situations participants face might be much more complex than what a single tip or text can address directly. Therefore, more comprehensive support mechanisms, such as an “Ask-The-Expert” or similar, would be more valuable.

It is also important to continue involving students in the content creation and gathering feedback on the program. Literature confirms as well that the participatory development of comparable interventions with specific target groups (eg, disadvantaged groups) often results in higher engagement regarding the usage of the “products” [24,25,52]. Furthermore, consideration could also be given to surveying teachers. On one hand, the support of teachers is essential for recruiting school classes, as they do not receive compensation for the lessons they provide for recruitment. In addition, their perception of the class during the ongoing SmartCoach program could be valuable.

### Strengths and Limitations

The main strength of this study is that comprehensive qualitative interviews were conducted using a structured and theoretically sound framework (thereby enhancing the validity of results)—the COM-B model and TDF framework—in a relatively large sample of adolescents to derive factors associated with engagement in a digital substance use prevention program among adolescents. This participatory approach ensures that the optimization suggestions are practical and aligned with user needs. In addition to the interviews, nonresponder analyses were conducted to assess the generalizability of the results (regarding the sample) and understand differences between participants and nonparticipants.

The use of qualitative methods, particularly the use of NVivo for thematic analysis, is another significant strength [31]. This approach allows for a deep, nuanced understanding of participant experiences and perspectives, which is often not achievable through quantitative methods alone. The process is time-intensive and requires meticulous attention to detail, reflecting a high level of commitment and expertise. In general, qualitative research of this nature is relatively rare in the setting of the present research area, but is crucial for complementing quantitative findings identified in the primary study [36]. It adds depth to the understanding of engagement factors and provides rich, context-specific insights that can drive more effective program improvements.

At the same time, some limitations of this study should also be considered. First, it was challenging to reach students by phone to conduct the qualitative interviews. After the third attempt at contact, many still could not be reached, and only a few responded to the subsequent text message requesting a proposed appointment. As a result, 4 of 10 (41%) students could not be reached and thus were not interviewed. This limits the representativeness and generalizability of the results. In this regard, the results of the nonresponder analysis indicated a significant association between participation in the interview and gender as well as the number of interactions in the SmartCoach program. Students who did not participate in the interview were more likely to be male and showed lower program engagement. This suggests a bias in the results reported toward female students with higher program engagement.

Second, the study sample consists of adolescents from specific regions in Switzerland, which may limit the generalizability of the findings to other populations or cultural contexts. Variations in substance use norms, digital health literacy, and educational systems across different regions and countries could impact the applicability of the results.

Third, socially desirable responses could not be entirely ruled out, despite a section that addressed this issue during the validation of the questionnaire. Fourth, qualitative research is characterized by subjectivity. On one hand, interviews are a form of self-disclosure and can therefore be distorted by phenomena such as memory distortion, personal perspectives, or the aforementioned social desirability. On the other hand, research questions, theoretical approaches, methods, and interpretations of results are also influenced to some extent by the subjectivity of the researchers [53]. This leads to a limitation of objectivity.

Finally, the timing of the study may have influenced engagement levels, especially if conducted during periods of heightened stress or significant events (eg, exam periods, pre-Christmas, and holidays). These temporal factors were not controlled for, which could affect the consistency of engagement data.

### Conclusion

This study identified key factors influencing engagement in a digital life skills training program for addiction prevention in adolescents. By addressing the identified barriers (eg, “Environmental context and resources” by adjusting the timing of messages and “Memory, attention, and decision process” by implementing reminders) and enhancing promoting factors such as “Beliefs about positive consequences,” digital interventions such as SmartCoach can achieve higher engagement rates and, ultimately, greater effectiveness in preventing substance use among adolescents. The insights gained contribute to the broader understanding of engagement in digital health interventions and offer a concrete approach for the continued development of SmartCoach and similar programs. The combination of these findings derived from qualitative research, in combination with results from quantitative predictor analysis [36], might also be of interest to optimize and tailor intervention programs for specific subgroups, for example, those defined by specific social stratifying factors or different levels of program engagement.

## Abbreviations

COM-B: Capability, Opportunity, Motivation-Behavior
JARS: Journal Article Reporting Standards for Qualitative Research
mHealth: mobile health
TDF: Theoretical Domains Framework
WHO: World Health Organization
